# Identification of differentially expressed genes in actinic keratosis samples treated with ingenol mebutate gel

**DOI:** 10.1371/journal.pone.0232146

**Published:** 2020-05-15

**Authors:** Sonia Segura, Alejandra Gadea, Lara Nonell, Evelyn Andrades, Silvia Sánchez, Ramon Pujol, Inmaculada Hernández-Muñoz, Agustí Toll

**Affiliations:** 1 Department of Dermatology, Hospital del Mar, Parc de Salut Mar, Barcelona, Spain; 2 Group of Inflamatory and Neoplasic Dermatological Diseases, IMIM (Hospital del Mar Medical Research Institute), Barcelona, Spain; 3 Centre National de la Recherche Scientifique (CNRS), Centre de Recherche Paul Pascal (CRPP), Université de Bordeaux, Pessac, France; 4 MARGenomics, IMIM (Hospital del Mar Medical Research Institute), Barcelona, Spain; 5 Department of Dermatology, Hospital Clínic de Barcelona, University of Barcelona, Barcelona, Spain; 6 Institut d’Investigacions Biomèdiques August Pi i Sunyer (IDIBAPS), Barcelona, Spain; University of Minnesota, UNITED STATES

## Abstract

Actinic keratosis is a common skin disease that may progress to invasive squamous cell carcinoma if left untreated. Ingenol mebutate has demonstrated efficacy in field treatment of actinic keratosis. However, molecular mechanisms on ingenol mebutate response are not yet fully understood. In this study, we evaluated the gene expression profiles of actinic keratosis lesions before and after treatment with ingenol mebutate using microarray technology. Actinic keratoses on face/scalp of 15 immunocompetent patients were identified and evaluated after treatment with topical ingenol mebutate gel 0.015%, applied once daily for 3 consecutive days. Diagnostic and clearance of lesions was determined by clinical, dermoscopic, and reflectance confocal microscopy criteria. Lesional and non-lesional skin biopsies were subjected to gene expression analysis profiled by Affymetrix microarray. Differentially expressed genes were identified, and enrichment analyses were performed using STRING database. At 8 weeks post-treatment, 60% of patients responded to ingenol mebutate therapy, achieving complete clearance in 40% of cases. A total of 128 differentially expressed genes were identified following treatment, and downregulated genes (114 of 128) revealed changes in pathways important to epidermal development, keratinocyte differentiation and cornification. In responder patients, 388 downregulated genes (of 450 differentially expressed genes) were also involved in development/differentiation of the epidermis, and immune system-related pathways, such as cytokine and interleukin signaling. Cluster analysis revealed two relevant clusters showing upregulated profile patterns in pre-treatment actinic keratoses of responders, as compared to non-responders. Again, differentially expressed genes were mainly associated with cornification, keratinization and keratinocyte differentiation. Overall, the present study provides insight into the gene expression profile of actinic keratoses after treatment with ingenol mebutate, as well as identification of genetic signatures that could predict treatment response.

## Introduction

Actinic keratosis (AK) is a common skin disease characterized by thick, scaly, cutaneous lesions on chronic sun-exposed areas, and histologically by atypical keratinocytes extending to the basal layer of the epidermis, that may progress to invasive squamous cell carcinoma (SCC) if left untreated [1,2]. The risk to develop a cutaneous SCC is approximately 0.03–20% per year for any single lesion [3–6], and the risk of malignant progression for a patient affected by multiple AK lesions has been estimated within a wide range between 0.15% and 80% [7]. The highest malignant transformation rates are found in males, elderly patients, individuals with phototypes I and II, human papillomavirus infection, a family history of skin cancer, and in immune-suppressed individuals [8–10]. However, despite the advances in the recognition of clinic, dermoscopic and histologic patterns, it is not yet possible to predict which lesions will advance to SCC [11]. For this reason, an early diagnosis and effective treatment are recommended.

There are many variables that may influence the therapy choice in an individual, such as the number and location of lesions, age and immunological status of the patient, personal history of previous skin lesions and previous treatments [11]. Nevertheless, since AKs are usually multiple and often subclinical, a field-directed approach is suggested to remove clinically visible as well as non-visible lesions within the treatment area to prevent the development to invasive SCC [12].

Among the topical field therapies available, ingenol mebutate (IM) has demonstrated to be an ef-fective and well tolerated option for the treatment of AK, with a simple dosing regimen and a short treating course [13,14]. However, the precise mechanism of action by which the drug induces cell death is not fully understood, neither are the molecular mechanisms involved in the lack of response to IM.

Herein, we report the results of a prospective study investigating the transcriptomic profile of AK lesions before and after treatment with IM gel. Our study included clinical and dermoscopic evaluations together with a monitoring of the histological patterns of AKs by *in vivo* reflectance confocal microscopy (RCM), a non-invasive imaging technique. We also performed a comparative analysis of gene expression profiles of AKs responsive and unresponsive to the study treatment, in order to gain a better understanding of the molecular mechanisms involved and to identify potential genetic signatures of response to IM therapy.

## Materials and methods

### Study design and patient population

This was a single-center, single-arm, prospective observational study conducted in Hospital del Mar in Barcelona from April 2016 to April 2018, to evaluate the mRNA expression profile of AKs before and after treatment with IM. We prospectively enrolled fifteen immunocompetent adult patients aged ≥ 18 years, who were clinically diagnosed with at least one AK (grade I-II, according to Olsen’s classification [15]) on the head and/or scalp larger than 8 mm in diameter. All patients were regularly being followed-up for previous history of non-melanoma skin cancer at the Skin Cancer Clinic of the Hospital del Mar. Patients could not have received any pharmacological treatment for AK within 6 months before inclusion. Subjects were excluded if they were pregnant, breastfeeding, or planning on becoming pregnant during the course of the trial. All individual participants received written and verbal information concerning the study, provided signed written informed consent, and allowed photographs of the selected treatment areas to be taken. Data confidentiality and anonymity will be ensured, according to the provisions of the Spanish Organic Law 3/2018, of December 5, on Personal Data Protection and Digital Rights, and the provisions of Regulation (EU) 2016/679 of the European Parliament and of the Council of 27 April 2016. The study was approved by the local ethics committee (Comité Ético de Investigación Clínica- Parc de Salut Mar, n° 2016/6597/I) and conducted in accordance with the Declaration of Helsinki (Fortaleza, Brazil, October 2013).

### Study assessments

At the initial screening visit (visit 0), participants provided informed consent, and study dermatologists collected demographic data and performed a clinical evaluation of the lesions on the head suggestive of AK. AK lesions larger than 8 mm in diameter were identified, evaluated, and subsequently monitored at the pre-treatment visit (visit 1), at 4±2 days after treatment (visit 2), and at 8±1 weeks after treatment (visit 3).

Clinical assessment of AKs and photographs with a digital camera (Canon Powershot G10®, Ōita, Japan) were made at each study visit. At visits 1 and 3, dermoscopic images (DermliteFoto®, 3Gen, San Juan Capistrano, CA, USA) and RCM images of the skin lesional sites were also obtained.

At visit 2, adverse events were evaluated and referred to as local skin reactions (LSR). The severity of LSR was graded subjectively from 0 to 4 (with higher numbers indicating greater severity), according to a 6-point local skin response scale including erythema, flaking/scaling, crusting, swelling (edema), vesiculation/pustulation, and erosion/ulceration. The composite LSR score represents the sum of the six individual scores for each patient, yielding a maximum composite score of 24 [16].

At visit 3 (end of study), diagnostic and clearance of AK lesions was determined according to the clinical and dermoscopic criteria [17] in combination with RCM assessments. At this visit, the clinical response to IM-treatment was classified into two categories: responder patients, if they showed either complete disappearance of the lesion or a reduction in the clinical, dermoscopic and RCM grade; and non-responder patients, if they did not show any improvement of the lesion after treatment.

### Sample collection and treatment

At the pre-treatment visit, the AK treatment areas of all lesions were drawn with their exact location on a transparent sheet with the use of physical reference points as landmarks. Within each AK treatment area, two areas were selected for biopsy before and after treatment. One of them was biopsied at visit 1 (pre-treatment biopsy: AK-PRE), and the other one at visit 3 (post-treatment biopsy: AK-POST). A biopsy from the axilla or retro-auricular area (non-lesional non-sun-exposed skin: NSES) and a biopsy from a sun-exposed peri-lesional area (within 5 cm peri-lesional: SES) were also obtained at visit 1. Location of the selected treatment areas as well as biopsy locations were verified by referencing the transparency on which they were mapped. All biopsies were 4-mm skin punch biopsies −performed under local anesthesia−, and were subjected to gene expression analysis profiled by Affymetrix microarray.

After performing AK-PRE biopsy, biopsy wounds were allowed to heal for two weeks. Then, participants were instructed to treat the healed skin area with IM gel 0.015% (Picato®, LEO Pharma, Ballerup, Denmark), applied once daily for 3 consecutive days in the affected area, covering both clinical AK and the area surrounding the lesions.

### *In vivo* reflectance confocal microscopy imaging

RCM imaging was performed by means of a commercially available reflectance mode confocal laser scanning microscope (Vivascope 3000®, Caliber ID, Rochester, New York, USA), following standard protocols described elsewhere [18]. Horizontal 750×750 microns images of the lesional skin were obtained at different epidermal layers and upper dermis throughout the lesion. Furthermore, vertical mapping using the Vivastack function (Vivastack®, Caliber ID, Rochester, New York, USA) was done in 5 μm-step series of images from the surface to a maximum depth of 200 μm, starting at the stratum corneum and continuing throughout the epidermis and papillary dermis in the areas of interest. Confocal criteria for AK already described in the literature were systematically evaluated. Grade of dysplasia in AKs was assessed according to the criteria reported by Pellacani et al [19].

### RNA extraction and purification

Biopsy samples were stabilized by immersion in RNAlater (Ambion) for 24 hours and further embedded in OCT before freezing. Total RNA from frozen OCT-embedded samples was extracted using mirVana miRNA Isolation kit (Ambion, Life Technologies, Thermo Fisher Scientific, Waltham, MA, USA), according to the manufacturer’s instructions. After extraction, purity and integrity of the RNA were assessed by spectrophotometry and nanoelectrophoresis using the NanoDrop ND-2000 spectrophotometer (NanoDrop Technologies, USA) and the Nano lab-on-a-chip assay for total eukaryotic RNA using Bioanalyzer 2100 (Agilent Technologies, USA), respectively. All samples had good purity and integrity and were subsequently used for microarray hybridization and qRT-PCR confirmation.

### Microarray analysis

For microarrays analysis, amplification and labeling were performed according to GeneChip™ WT PLUS Reagent kit (P/N 703174 2017) protocol from Thermo Fisher, and then hybridized to GeneChip Clariom S Human Array (Thermo Fisher) in a GeneChip Hybridization Oven 645. Washing and scanning were performed using the Expression Wash, Stain and Scan User Manual (P/N 702731 2017) from Thermo Fisher and the GeneChip System (GeneChip Fluidics Station 450 and GeneChip Scanner 3000 7G).

Microarray data analysis was performed in the R statistical environment (version 3.4.3) [20] using core, CRAN and Bioconductor packages.

The data have been deposited in NCBI’s Gene Expression Omnibus and are accessible through GEO Series accession number GSE142108.

#### Data preprocessing

After an exhaustive quality control, raw data were preprocessed using the Robust Multi-array Average (RMA) algorithm [21] in the *aroma*.*affymetrix* R package [22] using the three standard steps: background correction, quantile normalization and summarization step in a log_2_ base.

#### Differential expression analysis

Linear Models for Microarray Data (LIMMA) [23] was used to identify differentially expressed genes (DEGs) between conditions. A paired design was considered using function *duplicateCorrelation* of the *limma* R package [23]. Although analyses were adjusted for multiple comparisons using the false discovery rate procedure, genes for posterior analyses were selected having a p-value<0.05 and a log fold change │LogFC│≥0.5. Venn diagrams were generated using self-programmed functions available at https://github.com/margenomics.

#### Clustering method for microarray gene expression data

Behavior of genes in the four conditions (AK-PRE, AK-POST, NSES and SES) was studied using regression models through the R package “microarray Significant Profiles” (maSigPro) [24,25]. In particular, DEG between the following comparisons: AK-PRE and AK-POST, AK-PRE and SES and SES and NSES; were obtained using *limma* and included in *maSigPro* to identify groups of DEGs based on their evolution pattern for responders and non-responders to the study treatment. Clustering used function was *hclust* with correlation based distance and Ward linkage method. The median measure was selected to summarize gene expression at each node. Due to the variability in the cluster expression ranges, 15 clusters were selected to guarantee the correct election of the treatment response evolution patterns in the selected genes and clusters.

#### Protein-Protein Interaction (PPI) construction

The PPI networks were constructed using the Search Tool for the Retrieval of Interacting Genes (STRING) database [26,27]. Proteins are represented by nodes and interactions of pairwise proteins by edges. The identified clusters are mapped as different colors, which are assigned arbitrarily.

#### Functional and pathway enrichment analyses

To analyze the DEGs at the functional level, Gene Ontology (GO) [28]—in the biological process (BP) category—and Reactome pathway enrichment analyses [29] were performed using STRING software.

### Quantitative real-time reverse transcription–polymerase chain reaction (qRT-PCR)

qRT-PCR was performed for a number of genes of interest to validate microarray data. Complementary DNA (cDNA) was reverse transcribed from total RNA using the Transcriptor First Strand cDNA Synthesis kit (Roche Diagnostics, Mannheim, Germany). SYBR Green primers were purchased from Sigma-Aldrich. Based on the literature and on its stable expression in the array, the gene TBPL2 (TATA-Box Binding Protein Like 2) was used as a reference gene to normalize each sample. Quantitative PCRs were run on QuantStudio™ 12K Flex Real-Time PCR System using the SYBR Green select PCR master mix (Applied Biosystems by Life Technologies, Austin, TX, USA). All samples were tested in triplicate. The relative changes in gene expression data were calculated by the 2^-ΔΔCt^ method [30]. Differences in gene expression were statistically assessed using the Mann–Whitney U test (p-value<0.05 considered as significant).

## Results

### Demographics, clinical characteristics, and therapeutic response

The study included 15 immunocompetent subjects with grade I-II AK, according to Olsen’s classification [15]. Demographic and clinical characteristics of patients, including AK severity at baseline, composite LSR score at visit 2, and therapeutic outcome of AKs are detailed in Table 1. All participants were male with a mean age of 79.1 years (range 69−91 years). The parietal area was the most commonly involved site (6/15, 40.0%), followed by the temple (5/15, 33.3%). Most patients had Fitzpatrick skin phototype II, and only four individuals had phototype III. All patients had androgenetic alopecia (Norwood-Hamilton classification of IV-VII). Lesion severity was Olsen grade I in 8 patients, and Olsen grade II in the 7 remaining cases. Nine of the 15 patients (9/15, 60%) responded to the study treatment either completely (6/15, 40%) or partially (3/15, 20%).

**Table 1:** Demographic and clinical characteristics of 15 immunocompetent patients with actinic keratoses and their therapeutic response to ingenol mebutate gel at 8 weeks post-treatment.

Sample name	Age (years)	Gender	Lesion location	Olsen grade	Fitzpatrick skin phototype	Composite LSR score^a^	Therapeutic response^b^
**AK1**	91	M	Temple	I	II	6	1
**AK2**	84	M	Parietal area	I	II	6	0
**AK3**	76	M	Temple	II	II	3	1
**AK4**	72	M	Malar area	I	II	6	1
**AK5**	77	M	Forehead	I	II	5	1
**AK6**	79	M	Forehead	II	II	6	1
**AK7**	85	M	Parietal area	II	II	9	0
**AK8**	83	M	Parietal area	I	II	5	1
**AK9**	78	M	Temple	I	II	13	0
**AK10**	69	M	Parietal area	I	II	3	0
**AK11**	72	M	Temple	II	III	7	0
**AK12**	73	M	Temple	II	III	9	0
**AK13**	87	M	Parietal area	II	III	13	1
**AK14**	89	M	Parietal area	I	III	9	1
**AK15**	72	M	Frontal area	II	II	9	1

AK: actinic keratosis; M: male; LSR: local skin response

^a^Composite LSR at visit 2. The composite LSR score (0–24) represents the sum of the scores graded from 0 to 4 on all six individual LSR categories

^b^therapeutic response at visit 3 (0 = not response; 1: response).

**Fig 1:**
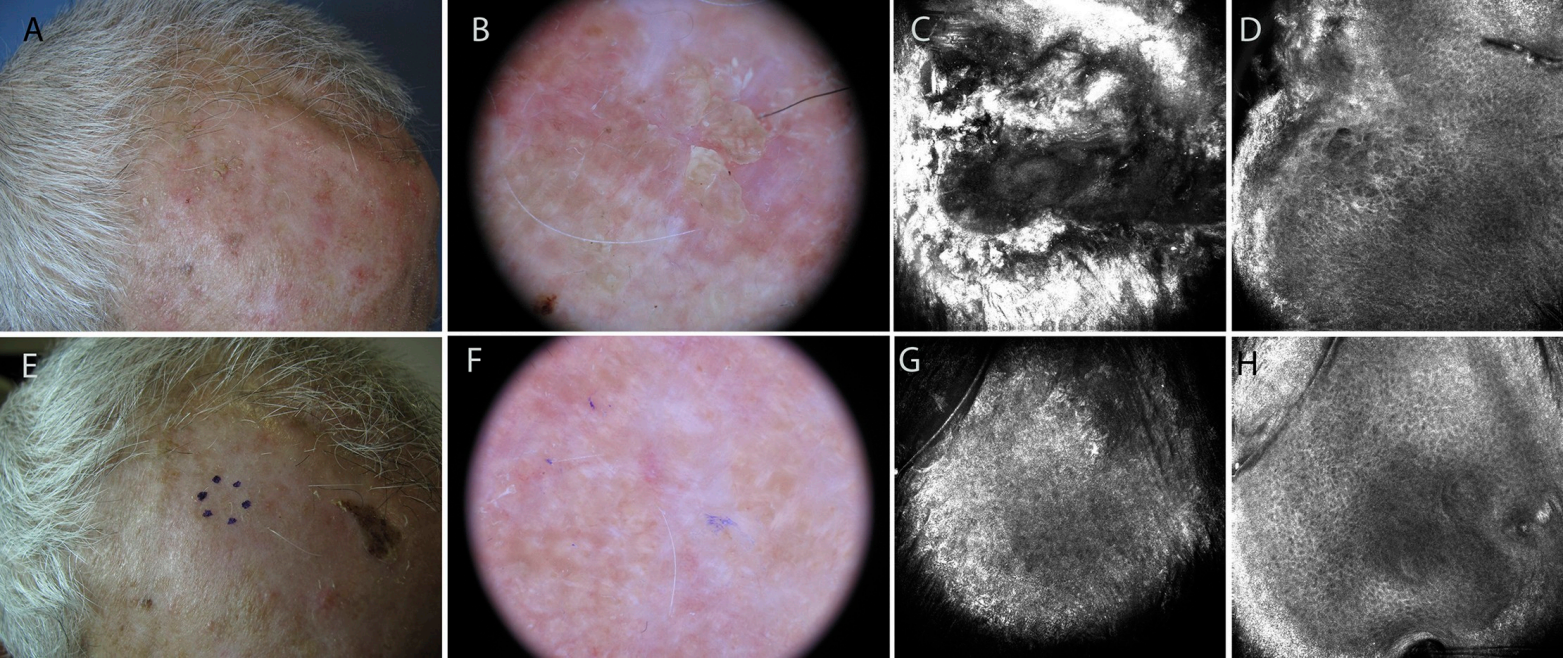
Responder patient treated with ingenol mebutate gel for a grade II actinic keratosis on the forehead (sample AK6). A: Clinical appearance of the lesion. B: Dermoscopic image showing background erythema and yellow scales with keratotic follicular openings. C: 750×750 μm confocal image at the stratum corneum showing marked hyperkeratosis. D: 750×750 μm confocal image at stratum spinosum demonstrating diffuse atypia of the keratinocytes with different sizes and shapes of the cells. E-F: Clinical and dermoscopic clearance of the lesion after ingenol mebutate treatment. G-H: 750×750 μm confocal images showing normal appearance of the skin.

Fig 1 shows the clinical, dermoscopic and confocal images of a responder patient (sample AK6) treated on the scalp, before and after the therapy. All patients presented at least one of the six LSRs evaluated. The mean composite score for each LSR was: erythema, 1.9 (range: 1–3); vesiculation/postulation, 1.5 (range: 0–3); flaking/scaling, 1.4 (range: 0–3); erosion/ulceration, 1.0 (range: 0–2); crusting, 1.0 (range: 0–2); and swelling, 0.3 (range: 0–1). The total mean composite LSR score derived from the overall analysis was 7.2 (range: 3–13).

### Microarray analysis

Whole genome gene expression profiling was performed on biopsies obtained from patients. We compared the expression profiles of: i) AK lesions before and after treatment with IM gel; ii) Post-treatment and pre-treatment AK lesions that responded to IM; and iii) AKs that responded and AKs that did not respond to the study treatment.

#### i) AK lesions before and after treatment with IM gel

A total of 128 DEGs were identified when comparing post-treatment with pre-treatment AK lesions. These DEGs included 114 downregulated and 14 upregulated genes. We performed Gene Ontology (GO) and Reactome pathway enrichment analyses for the 114 downregulated genes (Table 2). GO analysis showed that the strongly enriched terms were skin development, keratinocyte differentiation, keratinization and epidermis development. Reactome pathway analysis revealed formation of the cornified envelope and interleukin-36 pathway as that the most significantly enriched pathways. The downregulated DEGs assigned to these processes involved members of the *LCE1*/*LCE2*/*LCE3*/*LCE6* groups (late cornified envelope family), members of the *SPRR2*/*SPRR3* groups (small proline-rich protein genes), *KRT6C*/*KRT14*/*KRT80* (keratin genes), and *PI3* (peptidase inhibitor 3 gene), among others.

**Table 2:** Gene Ontology and Reactome pathway enrichment analyses of 114 downregulated differentially expressed genes in post-treatment versus pre-treatment actinic keratosis samples.

	Biological Process (GO)		
GO-term	Description	FDR	Downregulated matching proteins
GO:0030216	Keratinocyte differentiation	2.98E-24	CASP14, CNFN, CSTA, EREG, HRNR, IVL, KLK13, KRT14, KRT6C, KRT80, LCE1A, LCE1F, LCE2A, LCE2B, LCE2C, LCE2D, LCE3D, LCE3E, LCE6A, LIPN, LOR, PI3, PIP5K1A, SPRR2B, SPRR2D, SPRR2E, SPRR2F, SPRR2G, SPRR3
GO:0043588	Skin development	2.98E-24	ALOX12B, CASP14, CNFN, CSTA, EREG, ERRFI1, GRHL3, HRNR, IVL, KLK13, KRT14, KRT6C, KRT80, LCE1A, LCE1F, LCE2A, LCE2B, LCE2C, LCE2D, LCE3D, LCE3E, LCE6A, LIPN, LOR, PI3, PIP5K1A, SPRR2B, SPRR2D, SPRR2E, SPRR2F, SPRR2G, SPRR3
GO:0031424	Keratinization	6.89E-24	CASP14, CNFN, CSTA, HRNR, IVL, KLK13, KRT14, KRT6C, KRT80, LCE1A, LCE1F, LCE2A, LCE2B, LCE2C, LCE2D, LCE3D, LCE3E, LCE6A, LIPN, LOR, PI3, SPRR2B, SPRR2D, SPRR2E, SPRR2F, SPRR2G, SPRR3
GO:0008544	Epidermis development	8.84E-24	BNC1, CASP14, CNFN, CSTA, EREG, FABP5, GRHL3, HRNR, IVL, KLK13, KRT14, KRT6C, KRT80, LCE1A, LCE1F, LCE2A, LCE2B, LCE2C, LCE2D, LCE3D, LCE3E, LCE6A, LIPN, LOR, PI3, PIP5K1A, SPRR2B, SPRR2D, SPRR2E, SPRR2F, SPRR2G, SPRR3
GO:0018149	Peptide cross-linking	3.02E-20	CSTA, IVL, LCE1A, LCE1F, LCE2A, LCE2B, LCE2C, LCE2D, LCE3D, LCE3E, LOR, PI3, SPRR2B, SPRR2D, SPRR2E, SPRR2F, SPRR3
	**Reactome pathways**		
Pathway	Description	FDR	Downregulated matching proteins
HSA-6809371	Formation of the cornified envelope	4.09E-27	CASP14, CSTA, IVL, KLK13, KRT14, KRT6C, KRT80, LCE1A, LCE1F, LCE2A, LCE2B, LCE2C, LCE2D, LCE3D, LCE3E, LCE6A, LIPN, LOR, PI3, SPRR2B, SPRR2D, SPRR2E, SPRR2F, SPRR2G, SPRR3
HSA-9014826	Interleukin-36 pathway	9.60E-04	IL36B, IL36G, IL36RN
HSA-446652	Interleukin-1 family signaling	7.80E-03	IL1RN, IL36B, IL36G, IL36RN, MYD88, S100A12
HSA-5362517	Signaling by retinoic acid	7.80E-03	FABP5, PDK1, PPARD, SDR16C5
HSA-6798695	Neutrophil degranulation	1.80E-02	FABP5, GGH, HPSE, HRNR, PNP, PRSS3, S100A12, SERPINB3, SLPI, TCN1

GO: Gene Ontology; FDR: false discovery rate.

**Fig 2:**
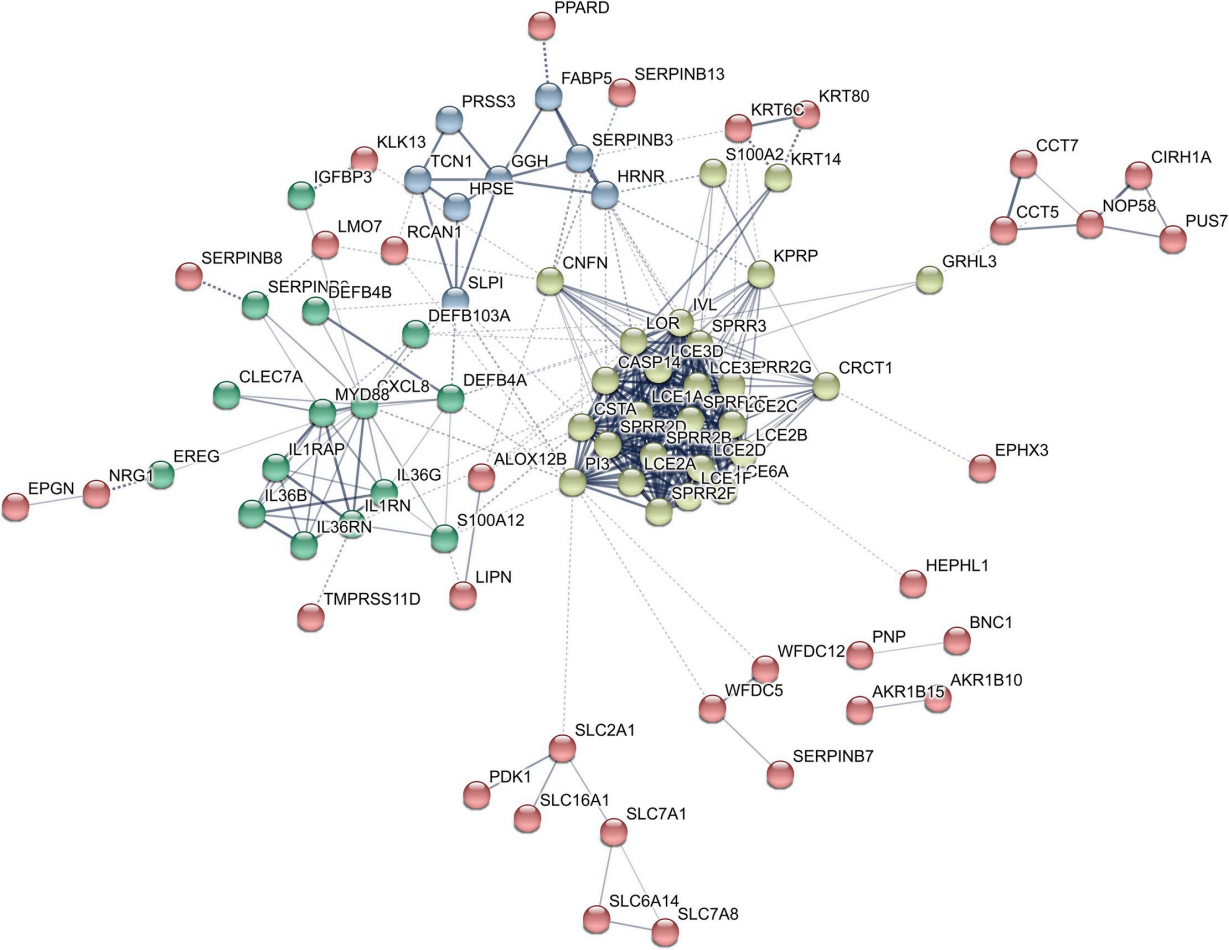
Constructed protein-protein interaction network of 114 downregulated genes in post-treatment versus pre-treatment actinic keratosis samples. Nodes represent proteins and different line intensities denote the type of evidence for the interaction. Statistical analysis results for the network: number of nodes: 114, number of edges: 355; average node degree: 6.23; average local clustering coefficient: 0.48; expected number of edges: 35; PPI enrichment p-value < 1E-16.

The PPI network of the 114 downregulated genes is shown in Fig 2, and a detailed list of proteins contained in the main clusters of the PPI network is summarized in S1 Table (see S1 Table). Most proteins belonging to the yellow cluster were involved in epidermal development, keratinocyte differentiation and cornification. Proteins of the green cluster mainly included cytokines, whereas most proteins of the blue cluster were enzymes, such as serine protease 3, gamma-glutamyl hydrolase and heparanase.

#### ii) Post-treatment and pre-treatment AK lesions that responded to IM

There were 450 DEGs between post-treatment and pre-treatment AKs responsive to IM, with 388 downregulated and 62 upregulated genes. The downregulated genes were enriched in BPs terms associated with epidermis development, keratinocyte differentiation, cornification, keratinization and epidermal cell differentiation (Table 3). The most significantly enriched pathways were the formation of the cornified envelope and immune system-related pathways, such as cytokine signaling, interleukin signaling, and neutrophil degranulation. Downregulated genes in these pathways included members of the *SPRR1*/*SPRR2*/*SPRR3* groups (small proline-rich protein genes), members of the keratin family (*KRT5*/*KRT6*/*KRT14*/*KRT16*/*KRT17*/*KRT19*), members of the *IL1*/*IL2*/*IL6-like cytokines*/*IL10* families (interleukin families), members of the *LCE2*/*LCE3* groups (late cornified envelope family), members of the *SERPINB* family (*SERPINB1*/*SERPINB2/SERPINB3*), members of the *S100* family (*S100A7*/ *S100A8*/*S100A9*/*S100A12*), *PI3* (peptidase inhibitor 3 gene), *CSTA* (cystatin-A gene) and *IVL* (involucrin gene).

**Table 3:** Gene Ontology and Reactome pathway enrichment analyses for 388 downregulated genes in post-treatment versus pre-treatment actinic keratoses that responded to ingenol mebutate gel.

	**Biological Process (GO)**		
GO-term	Description	FDR	Downregulated matching proteins
GO:0008544	Epidermis development	2.88E-18	ANXA1, BNC1, CASP14, CDH3, CNFN, COL17A1, CRABP2, CSTA, DSC2, DSG3, EREG, FABP5, GRHL3, HDAC1, HRNR, IVL, JUP, KLK13, KRT14, KRT16, KRT17, KRT19, KRT5, KRT6A, KRT6B, KRT6C, KRT80, LAMA3, LAMB3, LCE2A, LCE3A, LCE3C, LCE3D, LCE3E, PI3, PIP5K1A, RPTN, S100A7, SFN, SPRR1A, SPRR1B, SPRR2A, SPRR2B, SPRR2D, SPRR2E, SPRR2F, SPRR2G, SPRR3
GO:0030216	Keratinocyte differentiation	2.94E-18	ANXA1, CASP14, CDH3, CNFN, CSTA, DSC2, DSG3, EREG, HRNR, IVL, JUP, KLK13, KRT14, KRT16, KRT17, KRT19, KRT5, KRT6A, KRT6B, KRT6C, KRT80, LCE2A, LCE3A, LCE3C, LCE3D, LCE3E, PI3, PIP5K1A, RPTN, S100A7, SFN, SPRR1A, SPRR1B, SPRR2A, SPRR2B, SPRR2D, SPRR2E, SPRR2F, SPRR2G, SPRR3
GO:0009913	Epidermal cell differentiation	2.32E-17	ANXA1, CASP14, CDH3, CNFN, CSTA, DSC2, DSG3, EREG, HDAC1, HRNR, IVL, JUP, KLK13, KRT14, KRT16, KRT17, KRT19, KRT5, KRT6A, KRT6B, KRT6C, KRT80, LCE2A, LCE3A, LCE3C, LCE3D, LCE3E, PI3, PIP5K1A, RPTN, S100A7, SFN, SPRR1A, SPRR1B, SPRR2A, SPRR2B, SPRR2D, SPRR2E, SPRR2F, SPRR2G, SPRR3
GO:0031424	Keratinization	2.32E-17	CASP14, CDH3, CNFN, CSTA, DSC2, DSG3, HRNR, IVL, JUP, KLK13, KRT14, KRT16, KRT17, KRT19, KRT5, KRT6A, KRT6B, KRT6C, KRT80, LCE2A, LCE3A, LCE3C, LCE3D, LCE3E, PI3, RPTN, SFN, SPRR1A, SPRR1B, SPRR2A, SPRR2B, SPRR2D, SPRR2E, SPRR2F, SPRR2G, SPRR3
GO:0070268	Cornification	2.32E-17	CASP14, CSTA, DSC2, DSG3, IVL, JUP, KLK13, KRT14, KRT16, KRT17, KRT19, KRT5, KRT6A, KRT6B, KRT6C, KRT80, LCE3D, PI3, RPTN, SPRR1A, SPRR1B, SPRR2A, SPRR2B, SPRR2D, SPRR2E, SPRR2F, SPRR2G, SPRR3
	**Reactome pathways**		
Pathway	Description	FDR	Downregulated matching proteins
HSA-6809371	Formation of the cornified envelope	2.01E-20	CASP14, CSTA, DSC2, DSG3, IVL, JUP, KLK13, KRT14, KRT16, KRT17, KRT19, KRT5, KRT6A, KRT6B, KRT6C, KRT80, LCE2A, LCE3A, LCE3C, LCE3D, LCE3E, PI3, RPTN, SPRR1A, SPRR1B, SPRR2A, SPRR2B, SPRR2D, SPRR2E, SPRR2F, SPRR2G, SPRR3
HSA-9014826	Immune system	8.47E-08	L36RN, IL6, IL7R, JUNB, JUP, KIF18A, KIF20A, KIF23, KPNA2, LCN2, MALT1, MCL1, MMP1, MT2A, MYC, MYD88, NDC1, PGAM1, PI3, PKM, PLAC8, PLAUR, PNP, POLR3G, PRSS3, PSMA6, PSMD11, PSME3, PTGS2, PYGL, S100A12, S100A7, S100A7A, S100A8, S100A9, SEC31A, SELL, SERPINB1, SERPINB2, SERPINB3, SLC2A3, SLPI, TCN1, THEM4, TIMP1, TUBA4A, TXNDC5
HSA-1280215	Cytokine signaling in immune system	1.98E-06	ABCE1, ANXA1, CDKN1A, CFL1, CNN2, CXCL8, DUSP6, EGR1, EIF4A1, GBP6, HIF1A, HLA-DPB1, HSPA8, IFI27, IL1B, IL1RN, IL20, IL2RA, IL36G, IL36RN, IL6, IL7R, JUNB, KPNA2, LCN2, MCL1, MMP1, MT2A, MYC, MYD88, NDC1, PSMA6, PSMD11, PSME3, PTGS2, S100A12, SERPINB2, TIMP1
HSA-6798695	Neutrophil degranulation	2.81E-06	CDA, CNN2, CTSC, FABP5, GLIPR1, GMFG, GSTP1, HPSE, HRNR, HSPA8, JUP, LCN2, PGAM1, PKM, PLAC8, PLAUR, PNP, PRSS3, PSMD11, PYGL, S100A12, S100A7, S100A8, S100A9, SELL, SERPINB1, SERPINB3, SLC2A3, SLPI, TCN1, TXNDC5
HSA-5362517	Signaling by interleukins	6.40E-06	ANXA1, CDKN1A, CFL1, CNN2, CXCL8, DUSP6, HIF1A, HSPA8, IL1B, IL1RN, IL20, IL2RA, IL36G, IL36RN, IL6, IL7R, JUNB, LCN2, MCL1, MMP1, MYC, MYD88, PSMA6, PSMD11, PSME3, PTGS2, S100A12, SERPINB2, TIMP1

**Fig 3:**
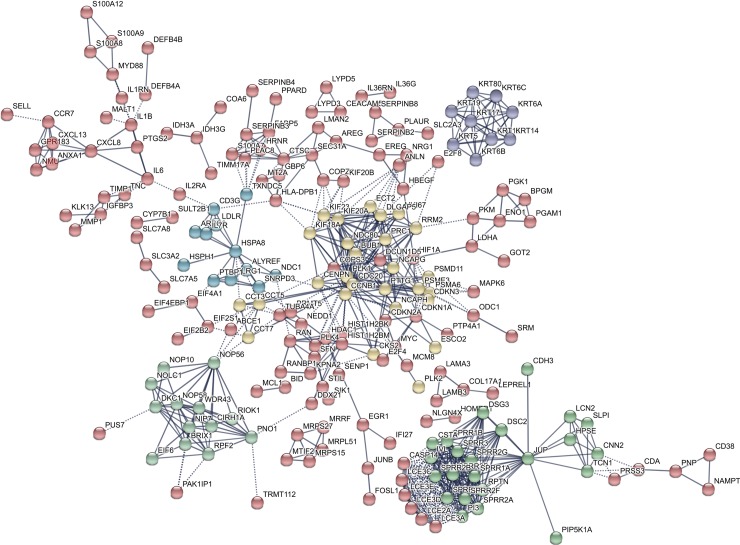
Constructed protein-protein interaction network of 388 downregulated genes in post-treatment versus pre-treatment actinic keratosis samples that responded to ingenol mebutate gel. Nodes represent proteins and different line intensities denote the type of evidence for the interaction. Statistical analysis results for the network: number of nodes: 388, number of edges: 715; average node degree: 3.69; average local clustering coefficient: 0.418; expected number of edges: 317; PPI enrichment p-value < 1E-16.

Fig 3 shows the PPI network of the 388 downregulated genes. The two most significant clusters were the yellow and the green ones. Proteins in both clusters are summarized in S2 Table (see S2 Table). Proteins of the yellow cluster were mainly involved in immune system-related pathways, whereas proteins of the green cluster were implicated in development/differentiation of the epidermis.

GO and Reactome pathway analyses results for the 62 upregulated DEGs are shown in S3 Table (see S3 Table). Taken together, both analyses revealed terms associated with extracellular structure and matrix organization, collagen fibril organization, and keratan sulfate catabolic process. The most significantly upregulated genes assigned to these pathways included collagen genes (*COL1A2*/*COL12A1*/*COL14A1*), lysyl oxidase gene (*LOX*) and dermatopontin (*DPT*).

The PPI network is shown in S1 Fig (see S1 Fig).

**Fig 4:**
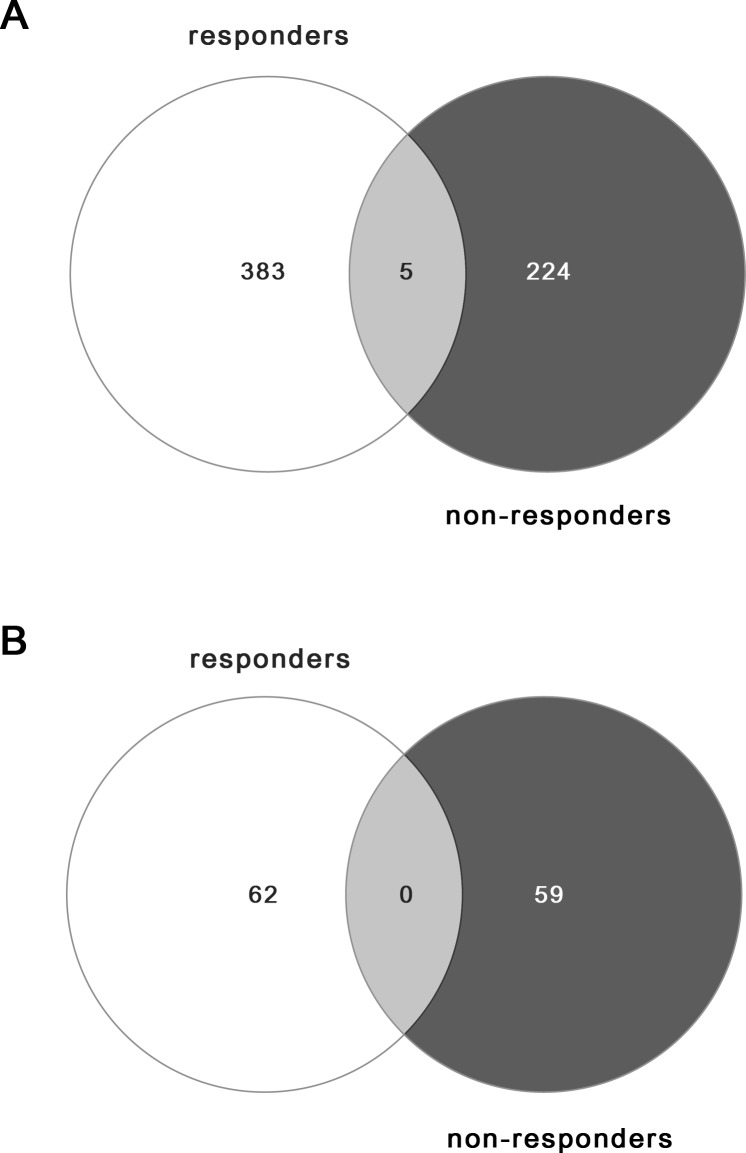
Venn diagrams illustrating the number of common and specific differentially expressed genes in IM treatment responders and non-responders. A. Downregulated genes. B. Upregulated genes.

Venn diagrams were also performed with the lists of DEGs obtained for AK lesions that responded to treatment with IM, compared to AK lesions that did not respond (Fig 4).

The Venn diagrams show the number of common and specific DEGs in each group. The light grey areas highlight the intersection between groups, where 5 downregulated DEGs and 0 upregulated genes were shared. In the responders’ group, 383 downregulated and 62 upregulated genes were uniquely expressed, while 224 downregulated and 59 upregulated genes were uniquely expressed in the non-responders’ group.

The lists of both downregulated and upregulated genes represented with Venn diagrams are presented in S1 Appendix and S2 Appendix, respectively.

#### iii) AKs that responded and AKs that did not respond to the study treatment (Cluster analysis)

**Fig 5:**
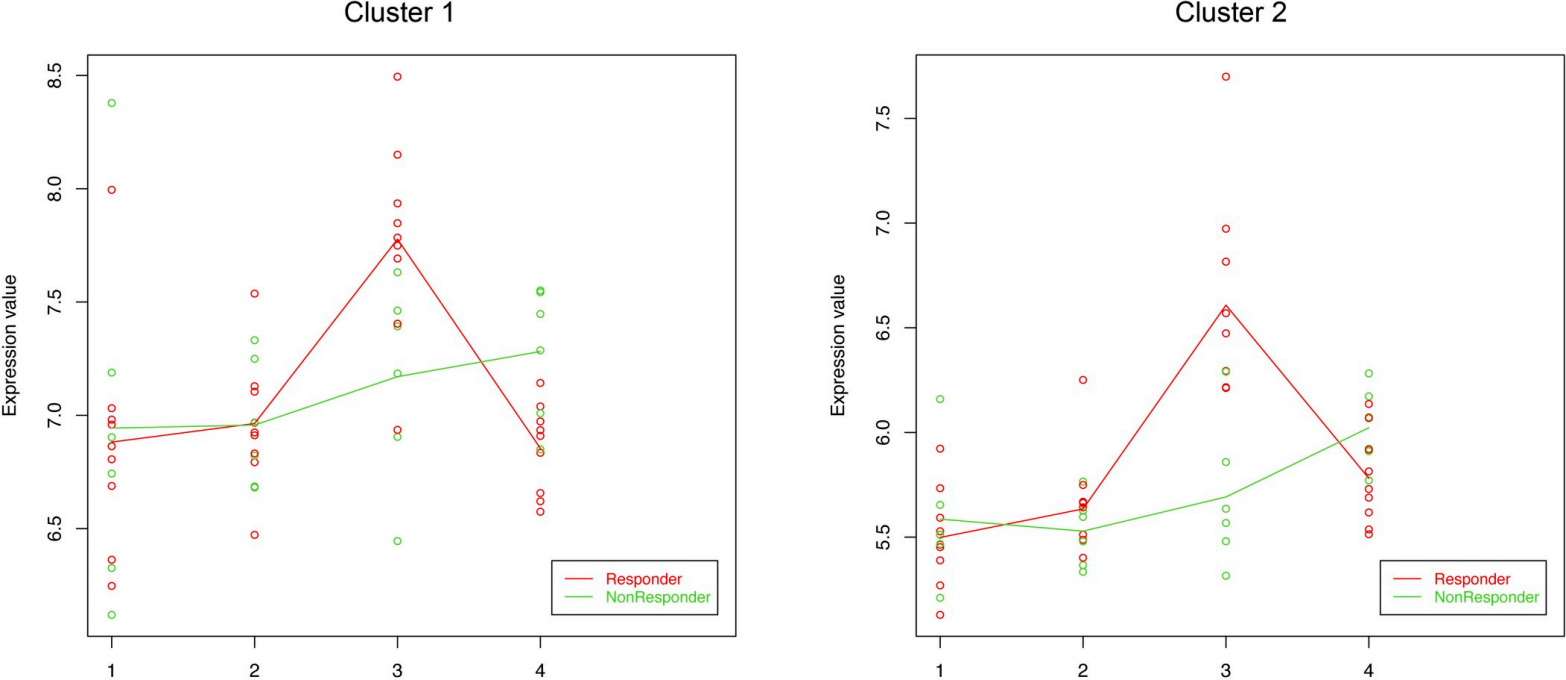
Cluster analysis. Each plot shows the average expression profile of all genes in the cluster by condition. The expression values of the clustered genes are represented in red for responder patients and in green for non-responders. Solid lines indicate the average value of gene expression at each condition for each group (responders and non-responders). 1: non-lesional non-sun-exposed skin samples; 2: sun-exposed peri-lesional skin samples; 3: pre-treatment actinic keratosis samples; 4: post-treatment actinic keratosis samples.

DEGs identified using maSigPro were grouped into fifteen clusters, showing distinct expression profiles across the four samples. Two of the clusters (clusters 1 and 2) showed a different pattern of expression for responder and non-responder patients before treatment with IM (AK-PRE samples), as compared to non-AK samples (both NSES and SES) and post-treatment samples (AK-POST) (Fig 5).

Both clusters shared similar expression patterns, but at different intensity levels. These two clusters aided in discriminating those genes that were only upregulated in IM-responders. The corresponding DEGs as well as GO and Reactome pathway analyses are shown in Table 4. The most enriched BPs terms and pathways were associated with cornification, keratinization, keratinocyte differentiation and peptide cross-linking. Other enriched terms were related to the immune system category, including defense response, immune response and leukocyte migration. Upregulated genes in IM-responders attributed to these pathways involved members of the *SPRR1*/*SPRR2* groups (small proline-rich protein genes), members of the *LCE3* group (late cornified envelope family), members of the keratin family (*KRT6*/ *KRT17*/*KRT19*), *PI3* (peptidase inhibitor 3 gene), *KLK13* (kallikrein-related peptidase 13), members of the *S100* family (*S100A7*/ *S100A8*/*S100A9*/*S100A12*), members of the CXCL chemokine family (*CXCL8*/*CXCL13*), and members of the beta defensins family (*DEFB4A*/*DEFB4B*/*DEFB103A*), among others.

**Table 4:** Gene Ontology and Reactome pathway enrichment analyses for clusters 1 and 2.

		Biological Process (GO)		
Cluster	GO-term	Description	FDR	Matching proteins
Cluster 1	**GO:0070268**	Cornification	5.16E-19	DSC2, KLK13, KRT17, KRT19, KRT6A, KRT6B, KRT6C, LCE3D, PI3, SPRR1A, SPRR1B, SPRR2A, SPRR2B, SPRR2D, SPRR2E, SPRR2F, SPRR2G
	**GO:0030216**	Keratinocyte differentiation	5.72E-19	DSC2, KLK13, KRT17, KRT19, KRT6A, KRT6B, KRT6C, LCE3A, LCE3C, LCE3D, LCE3E, PI3, S100A7, SPRR1A, SPRR1B, SPRR2A, SPRR2B, SPRR2D, SPRR2E, SPRR2F, SPRR2G
	**GO:0031424**	Keratinization	5.72E-19	DSC2, KLK13, KRT17, KRT19, KRT6A, KRT6B, KRT6C, LCE3A, LCE3C, LCE3D, LCE3E, PI3, SPRR1A, SPRR1B, SPRR2A, SPRR2B, SPRR2D, SPRR2E, SPRR2F, SPRR2G
	**GO:0018149**	Peptide cross-linking	6.74E-15	LCE3A, LCE3C, LCE3D, LCE3E, PI3, SPRR1A, SPRR1B, SPRR2A, SPRR2B, SPRR2D, SPRR2E, SPRR2F
	**GO:0030855**	Epithelial cell differentiation	2.62E-14	CD24, DSC2, KLK13, KRT17, KRT19, KRT6A, KRT6B, KRT6C, LCE3A, LCE3C, LCE3D, LCE3E, PI3, RHCG, S100A7, SPRR1A, SPRR1B, SPRR2A, SPRR2B, SPRR2D, SPRR2E, SPRR2F, SPRR2G
Cluster 2	**GO:0002376**	Immune system process	2.84E-08	ANXA1, BPGM, C10orf99, C5AR1, CLEC4E, CXCL13, CXCL8, DEFB103A, DEFB4A, DEFB4B, EGR1, FABP5, FCGR1A, FPR1, GBP6, GNLY, GPR15, HIF1A, HRNR, IL6, INHBA, KYNU, MMP1, MT2A, NR4A3, PLAUR, PNP, POLR3G, S100A12, SELE, SLC7A11, TCN1, TXNDC5
	**GO:0006952**	Defense response	2.40E-07	ANXA1, C10orf99, C5AR1, CLEC4E, CXCL13, CXCL8, DEFB103A, DEFB4A, DEFB4B, EGR1, FCGR1A, FPR1, GBP6, GNLY, HIF1A, IL6, INHBA, KYNU, MT2A, POLR3G, PTGS2, S100A12, SELE
	**GO:0006950**	Response to stress	4.70E-07	ANXA1, C10orf99, C5AR1, CDH3, CLEC4E, CXCL13, CXCL8, DEFB103A, DEFB4A, DEFB4B, EGR1, ERRFI1, FCGR1A, FPR1, GBP6, GNLY, GPX2, HIF1A, IL6, INHBA, KYNU, MT2A, MYC, NR4A2, NR4A3, PLAUR, POLR3G, PTGS2, S100A12, SELE, SGK1, SLC7A11, SPRR3, STC1, TXNDC5, ZFP36
	**GO:0006955**	Immune response	4.70E-07	ANXA1, C5AR1, CLEC4E, CXCL13, CXCL8, DEFB103A, DEFB4A, DEFB4B, EGR1, FABP5, FCGR1A, FPR1, GBP6, GNLY, HRNR, IL6, KYNU, MT2A, NR4A3, PLAUR, PNP, POLR3G, S100A12, TCN1, TXNDC5
	**GO:0050900**	Leukocyte migration	1.18E-06	ANXA1, C10orf99, C5AR1, CXCL13, CXCL8, FPR1, GPR15, IL6, MMP1, S100A12, SELE, SLC7A11
		**Reactome pathways**		
Cluster	**GO-term**	Description	FDR	Matching proteins
Cluster 1	**HSA-6809371**	Formation of the cornified envelope	5.33E-24	DSC2, KLK13, KRT17, KRT19, KRT6A, KRT6B, KRT6C, LCE3A, LCE3C, LCE3D, LCE3E, PI3, SPRR1A, SPRR1B, SPRR2A, SPRR2B, SPRR2D, SPRR2E, SPRR2F, SPRR2G
	**HSA-1266738**	Developmental Biology	7.02E-09	CD24, DSC2, KLK13, KRT17, KRT19, KRT6A, KRT6B, KRT6C, LCE3A, LCE3C, LCE3D, LCE3E, PI3, SPRR1A, SPRR1B, SPRR2A, SPRR2B, SPRR2D, SPRR2E, SPRR2F, SPRR2G
	**HSA-6799990**	Metal sequestration by antimicrobial proteins	2.20E-06	S100A7, S100A7A, S100A8, S100A9
	**HSA-69278**	Cell cycle, mitotic	1.15E-05	AURKA, CCNB1, CDC20, CDKN2A, CENPN, KIF20A, KIF23, NCAPG, NCAPG2, NDC80, PTTG1, RRM2
	**HSA-68886**	M Phase	2.00E-04	CCNB1, CDC20, CENPN, KIF20A, KIF23, NCAPG, NCAPG2, NDC80, PTTG1
Cluster 2	**HSA-168256**	Immune system	2.14E-06	ANXA1, C5AR1, CD22, CLEC4E, CXCL8, DEFB4B, EGR1, FABP5, FCGR1A, FPR1, GBP6, GNLY, HIF1A, HRNR, IL20, IL6, MMP1, MT2A, MYC, PLAUR, PNP, POLR3G, PTGS2, S100A12, TCN1, TXNDC5
	**HSA-1280215**	Cytokine signaling in immune system	3.22E-05	ANXA1, CXCL8, EGR1, FCGR1A, FPR1, GBP6, HIF1A, IL20, IL6, MMP1, MT2A, MYC, PTGS2, S100A12
	**HSA-6785807**	Interleukin-4 and Interleukin-13 signaling	3.22E-05	ANXA1, CXCL8, HIF1A, IL6, MMP1, MYC, PTGS2
	**HSA-449147**	Signaling by interleukins	5.90E-04	ANXA1, CXCL8, FPR1, HIF1A, IL20, IL6, MMP1, MYC, PTGS2, S100A12
	**HSA-168249**	Innate Immune System	1.90E-03	C5AR1, CLEC4E, DEFB4B, FABP5, FCGR1A, FPR1, GNLY, HRNR, PLAUR, PNP, POLR3G, S100A12, TCN1, TXNDC5

GO: Gene Ontology; FDR: false discovery rate.

S2 Fig and S3 Fig show the PPI networks of the DEGs of clusters 1 and 2, respectively (see S2 and S3 Figs).

### Quantitative real-time reverse transcription–polymerase chain reaction (qRT-PCR) analysis

Four differentially expressed genes in responder versus non-responder patients (EPGN, PI3, GJB2 and SERPINB4) were validated by qRT-PCR. Boxplots generated on the log2-transformed data illustrate the variations in expression levels of these genes between the two groups (see S4 Fig). Downregulation of these genes in responders was confirmed, suggesting good reliability for the genes identified by the microarray study. Extensive confirmation of genes altered in expression was not possible because of limitations in the amount of biopsy material available.

## Discussion

AKs are well-established precancerous skin lesions caused by cumulative DNA damage from exposure to ultraviolet radiation, leading to alterations of gene expression profiles in the skin. These modifications induce changes in important pathways involved in cellular proliferation, inflammation, immunosuppression, cell survival, terminal differentiation, tissue remodeling and apoptosis [31,32]. Understanding these mechanisms of AK formation guides the basis behind the current available treatments for AK [33].

IM is the drug most recently introduced as a safe and effective therapeutic option for non-hyperkeratotic and non-hypertrophic AKs in adults [34,35]. It appears to have a dual mechanism of action, causing cell death in transformed keratinocytes as well as inducing an inflammatory reaction after topical application [36].

The main objective of the present study was to characterize the gene expression profiles of AK samples before and after field-directed therapy with topical IM gel 0.015% using microarray technology. With this research we also aimed to identify genetic signatures that could be associated to IM response.

Treatment with IM applied on the face/scalp of 15 immunocompetent patients was found to be well tolerated, and the adverse effects were mild, with low LSR scores. The levels and values of the LSR composite scores obtained were not associated to the treatment efficacy. These results are in accordance with previous research [37,38].

At 8 weeks post-treatment, 60% (9/15) of patients demonstrated a response to this therapy, achieving complete clearance in 40% (6/15) of cases. These findings are similar to those obtained by Lebwohl *et al*. [16], showing partial and complete response rates ─for facial and scalp AKs treated with IM─ of about 64% and 42%, respectively. Our study included not only clinical evaluation of AK lesions, but also dermoscopic and RCM assessments that provided more information than clinical evaluation alone. In fact, RCM is considered the only reliable noninvasive alternative for skin biopsy, with RCM findings showing excellent correlation with routine histology [39].

We performed microarray analysis on AK samples to identify common pathways involved in the molecular effects of IM on AKs. We used two well-known public pathway repositories (GO and Reactome databases) to perform enrichment analyses. These analyses can be carried out through the use of several gene annotation databases to detect which functional categories or pathways are enriched in a specific list of genes or proteins [40]. Overall, the results of the functional analyses obtained with GO database overlapped with the results of the Reactome pathway analyses.

After IM-treatment, DEGs mainly revealed changes related to epidermal development, keratinocyte differentiation and cornification. Specifically, late cornified envelope genes, small proline-rich protein genes and keratin genes showed a dramatic decrease in expression after treatment. In fact, most of these genes have been previously shown to be increased upon exposure to UV and/or in AK [41–43]. Thus, their downregulation following IM-treatment indicate that pathways modulating the differentiation and proliferation of epidermal keratinocytes were normalized after treatment with IM. These results are consistent with a former reported study, in which IM-treated AK lesions resulted in downregulation of genes revealing a skin-signature involved in keratinization, keratinocyte differentiation and development of the epidermis [44].

Similar results were obtained when analyzing samples responsive to treatment with IM: we identified 388 downregulated genes associated with epidermis development, keratinocyte differentiation, cornification, keratinization, epidermal cell differentiation and formation of the cornified envelope. Some of the downregulated DEGs were also linked to immune system-related pathways, such as cytokine signaling, interleukin signaling, and neutrophil degranulation. Among them, *S100* genes, which have been suggested to play a role in regulating the epidermal response to tissue injury, inflammation and disease [45], SERPINB genes, which are important in inflammation and immune system function [46], and interleukin genes, have been previously seen to be overexpressed in pretreatment AK lesions [43].

The 62 upregulated genes after IM-treatment in responder patients were predominantly involved in extracellular structure and matrix organization, and collagen fibril organization, which are indicators of wound healing in the skin [47,48]. These results suggest a potential contribution of IM to the remodeling of the extracellular matrix, favoring AKs healing process. In fact, one of the main collagen-stimulating factors during the wound healing process (TGF-β) has previously been shown to be significantly increased after treatment with IM gel, resulting in anti-aging effects for patients with multiple AK lesions [49]. In our study, we observed an increased expression of the *LOX* gene, which is known to play a critical role in skin tumor progression [50]

Altogether, these findings show that IM-treatment of AKs primarily results in global downregulation of gene expression impacting various biological processes, being epidermal development, keratinocyte differentiation and cornification the major processes represented.

The cluster analysis was performed to identify group of genes that clearly expressed differences in behavior between responder and non-responder patients among the four analyzed biopsy samples (AK-PRE, AK-POST, NSES and SES). This analysis revealed two relevant clusters that showed upregulated profile patterns in AK-PRE samples of IM-responder patients as compared to non-responder patients. Again, DEGs were mainly associated with cornification, keratinization, keratinocyte differentiation, peptide cross-linking and formation of the cornified envelope. In particular, associated genes to these pathways involved small proline-rich protein genes, the peptidase inhibitor 3 gene, a late cornified envelope gene, keratin genes, and a kallikrein-related peptidase gene. These genes have been found to be overexpressed in AK lesions [43]. In addition, KLK13 has been considered an independent favorable prognosis biomarker for several cancers, including skin cancer [51]. Other upregulated DEGs were related to the immune system category, including defense response, immune response and leukocyte migration. DEGs associated to these processes included *S100*, *CXCL* and *DEFB* genes. The increased expression of certain *S100* and *DEFB4* genes in AK samples has been previously documented [43]. Interestingly, the overexpression of chemokine CXCL8, which plays a central role in recruiting immune cells to inflamed skin, has been also observed after IM-treatment [44]. Overexpression of these genes evidences a higher induced immune response in AK lesions of responder patients as compared to non-responder patients. Thereby, patients with a higher immune response may be potentially more reactive to ingenol.

The results of this study should be considered in the context of the limitations, including the small number of samples employed in the microarray analysis, which could generate biased results. On the other side, although qRT-PCR is a sensitive and precise tool for microarray validation, the limitation of biopsy material that could be extracted from the skin tissues was low, which restricted the number of target genes that were validated. Therefore, further investigations with larger sample sizes would be required to confirm our findings.

## Conclusions

The results herein presented provide insight into the gene expression profile of AK samples after treatment with topical IM, as well as the biological processes involved. In addition, this is the first comparison of untreated skin samples (non-sun-exposed skin, sun-exposed skin, and lesional skin) and AK lesions treated with IM between responder and non-responder patients, leading to the identification of genetic signatures that could be correlated with the treatment response.

## Supporting information

S1 Table List of proteins contained in the most relevant clusters of the protein-protein interaction network of 114 downregulated differentially expressed genes in post-treatment versus pre-treatment actinic keratosis samples. (DOCX)

S2 Table List of proteins contained in the most relevant clusters of the protein-protein interaction network of 388 downregulated genes in post-treatment versus pre-treatment actinic keratoses that responded to ingenol mebutate gel. (DOCX)

S3 Table Gene Ontology and Reactome pathway enrichment analyses for 62 upregulated genes in post-treatment versus pre-treatment actinic keratoses that responded to ingenol mebutate gel. (DOCX)

S1 Fig Constructed protein-protein interaction network of 62 upregulated genes in post-treatment versus pre-treatment actinic keratosis samples that responded to ingenol mebutate gel. Nodes represent proteins and different line intensities denote the type of evidence for the interaction. Statistical analysis results for the network: number of nodes: 62, number of edges: 43; average node degree: 1.39; average local clustering coefficient: 0.331; expected number of edges: 7; PPI enrichment p-value < 1E-16.
(TIF)

S2 Fig Constructed protein-protein interaction network of upregulated genes in pre-treatment actinic keratosis samples of responder patients as compared to non-responder patients (cluster 1). Nodes represent proteins and different line intensities denote the type of evidence for the interaction. Statistical analysis results for the network: number of nodes: 72, number of edges: 336; average node degree: 9.33; average local clustering coefficient: 0.656; expected number of edges: 39; PPI enrichment p-value < 1E-16.
(TIF)

S3 Fig Constructed protein-protein interaction network of upregulated genes in pre-treatment actinic keratosis samples of responder patients as compared to non-responder patients (cluster 2). Nodes represent proteins and different line intensities denote the type of evidence for the interaction. Statistical analysis results for the network: number of nodes: 76, number of edges: 116; average node degree: 3.05; average local clustering coefficient: 0.44; expected number of edges: 37; PPI enrichment p-value < 1E-16.
(TIF)

S4 Fig RT-qPCR validation experiments of selected genes (EPGN, PI3, GJB2 and SERPINB4). The box plots show the log2 expression levels in responder and non-responder patients (p-values <0.05). Empty circles indicate mild outliers and asterisks extreme outliers.
(TIF)

S1 Appendix (XLSX)

S2 Appendix (XLSX)
