# Supplementary material for: Identification of differentially expressed genes in actinic keratosis samples treated with ingenol mebutate gel
Source: PLoS One. 2020 May 15;15(5):e0232146. doi: 10.1371/journal.pone.0232146 (PMC7228095; doi:10.1371/journal.pone.0232146)
Supplement: S1 Table — (DOCX) [file pone.0232146.s001.docx]

**S1 Table.** List of proteins contained in the most relevant clusters of the protein-protein interaction network of 114 downregulated genes in post-treatment actinic keratosis samples after ingenol mebutate therapy as compared to pre-treatment samples.

| **Cluster color** | **Protein name** | **Protein description** |
| --- | --- | --- |
| Yellow | CRCT1 | Cysteine-rich C-terminal protein 1 |
|  | CASP14 | Encodes a member of the cysteine-aspartic acid protease (caspase) family |
|  | KPRP | Keratinocyte proline-rich protein (it encodes a proline-rich skin protein possibly involved in keratinocyte differentiation) |
|  | LCE3E | Late cornified envelope protein 3E |
|  | LCE2B | Late cornified envelope protein 2B |
|  | LCE1A | Late cornified envelope protein 1A |
|  | SPRR2G | Small proline-rich protein 2G (component of the cornified envelope) |
|  | SPRR2D | Small proline-rich protein 2D (component of the cornified envelope) |
|  | LCE2A | Late cornified envelope protein 2A |
|  | PI3 | Peptidase inhibitor 3 (Neutrophil and pancreatic elastase-specific inhibitor of skin) |
|  | LCE3D | Late cornified envelope protein 3D |
|  | LCE6A | Late cornified envelope protein 6A |
|  | CNFN | Cornifelin (component of the cornified envelope) |
|  | SPRR2B | Small proline-rich protein 2B (component of the cornified envelope) |
|  | LCE1F | Late cornified envelope protein 1F |
|  | LOR | Loricrin (is part of a cluster of genes on chromosome 1 called the epidermal differentiation complex) |
|  | LCE2C | Late cornified envelope protein 2C |
|  | SPRR2F | Small proline-rich protein 2F (component of the cornified envelope) |
|  | CSTA | Cystatin-A (encodes a skin barrier cystein protease inhibitor) |
|  | KRT14 | Keratin 14 |
|  | LCE2D | Late cornified envelope protein 2D |
|  | GRHL3 | Prodifferentiation transcription factor grainyhead-like 3 (epidermal differentiation gene) |
|  | IVL | Involucrin (component of the cornified envelope) |
|  | S100A2 | S100 Calcium Binding Protein A2 |
|  | SPRR3 | Small proline-rich protein 3 (component of the cornified envelope) |
|  | SPRR2E | Small proline-rich protein 2E (component of the cornified envelope) |
| Green | DEFB103A | Beta-defensin 103A |
|  | S100A12 | S100 Calcium Binding Protein A12 |
|  | IL36RN | Interleukin 36 Receptor Antagonist |
|  | IL36G | Interleukin 36 Gamma |
|  | IL36B | Interleukin 36 Beta |
|  | MYD88 | MYD88 Innate Immune Signal Transduction Adaptor |
|  | CLEC7A | C-type lectin domain family 7 member A or Dectin-1 |
|  | IL1RAP | Interleukin 1 receptor accessory protein |
|  | DEFB4A | Defensin beta 4A |
|  | IL1RN | Interleukin 1 receptor antagonist |
|  | EREG | Epiregulin [secreted peptide hormone and member of the epidermal growth factor (EGF) family of proteins] |
|  | SERPINB2 | Serpin family B member 2 |
|  | CXCL8 | Interleukin-8 |
|  | IGFBP3 | Insulin-like growth factor-binding protein 3 |
|  | DEFB4B | Defensin beta 4A |
| Blue | TCN1 | Transcobalamin I (Vitamin B12 Binding Protein, R Binder Family) |
|  | FABP5 | Fatty acid-binding protein 5 |
|  | HRNR | Hornerin (a novel member of the "fused gene"-type cornified envelope precursor protein) |
|  | PRSS3 | Serine protease 3 (trypsinogen-IV) |
|  | GGH | Gamma-glutamyl hydrolase |
|  | SERPINB3 | Serpin family B member 3 |
|  | HPSE | Heparanase |
|  | SLPI | Secretory leukocyte peptidase inhibitor |
