# Supplementary material for: Identification of differentially expressed genes in actinic keratosis samples treated with ingenol mebutate gel
Source: PLoS One. 2020 May 15;15(5):e0232146. doi: 10.1371/journal.pone.0232146 (PMC7228095; doi:10.1371/journal.pone.0232146)
Supplement: S2 Table — (DOCX) [file pone.0232146.s002.docx]

**S2 Table.** List of proteins contained in the most relevant clusters of the protein-protein interaction network of 388 downregulated genes in post-treatment versus pre-treatment actinic keratoses that responded to ingenol mebutate gel.

| **Cluster color** | **Protein name** | **Protein description** |
| --- | --- | --- |
| Yellow | DLGAP5 | DLG associated protein 5 (Disks large-associated protein 5) |
|  | CDC20 | Cell division cycle protein 20 |
|  | PLK2 | Polo like kinase 2 |
|  | MKI67 | Marker of proliferation Ki-67 |
|  | CDKN3 | Cyclin-dependent kinase inhibitor 3 |
|  | CCNB1 | Cyclin B1 |
|  | NCAPG | Non-SMC condensin I complex subunit G |
|  | NDC80 | NDC80 kinetochore complex component |
|  | PRC1 | Protein regulator of cytokinesis 1 |
|  | BUB1 | BUB1 mitotic checkpoint serine/threonine kinase |
|  | CCT3 | Chaperonin containing TCP1 subunit 3 |
|  | KIF20A | Kinesin family member 20A |
|  | CCT5 | Chaperonin containing TCP1 subunit 5 |
|  | PSMA6 | Proteasome subunit alpha 6 |
|  | KIF18A | Kinesin family member 18A |
|  | PSME3 | Proteasome activator subunit 3 |
|  | KIF23 | Kinesin family member 23 |
|  | ECT2 | Epithelial cell transforming 2 |
|  | CKS2 | CDC28 protein kinase regulatory subunit 2 |
|  | ESCO2 | Establishment of sister chromatid cohesion N-acetyltransferase 2 |
|  | PLK1 | Polo like kinase 1 |
|  | CENPN | Centromere protein N |
|  | PSMD11 | Proteasome 26S subunit, non-atpase 11 |
|  | CCT7 | Chaperonin containing TCP1 subunit 7 |
|  | NCAPH | Non-SMC condensin I complex subunit H |
|  | PTTG1 | PTTG1 regulator of sister chromatid separation, securin |
|  | RRM2 | Ribonucleotide reductase regulatory subunit M2 |
| Green | TCN1 | Transcobalamin i (vitamin b12 binding protein, r binder family) |
|  | SPRR2G | Small proline-rich protein 2G (component of the cornified envelope) |
|  | SPRR2D | Small proline-rich protein 2D (component of the cornified envelope) |
|  | SPRR1B | Small proline-rich protein 1B |
|  | PI3 | Peptidase inhibitor 3 (Neutrophil and pancreatic elastase-specific inhibitor of skin) |
|  | JUP | Junction plakoglobin |
|  | DSC2 | Desmocollin 2 |
|  | HPSE | Heparanase |
|  | RPTN | Repetin |
|  | SLPI | Secretory leukocyte peptidase inhibitor |
|  | CDH3 | Cadherin 3 |
|  | PIP5K1A | Phosphatidylinositol-4-phosphate 5-kinase type 1 alpha |
|  | DSG3 | Desmoglein 3 |
|  | SPRR2B | Small proline-rich protein 2B (component of the cornified envelope) |
|  | SPRR2F | Small proline-rich protein 2F (component of the cornified envelope) |
|  | CSTA | Cystatin-A (encodes a skin barrier cystein protease inhibitor) |
|  | SPRR2A | Small proline-rich protein 2A (component of the cornified envelope) |
|  | CNN2 | Calponin 2 |
|  | IVL | Involucrin (component of the cornified envelope) |
|  | SPRR3 | Small proline-rich protein 3 (component of the cornified envelope) |
|  | SPRR1A | Small proline rich protein 1A |
|  | LCN2 | Lipocalin 2 |
|  | SPRR2E | Small proline-rich protein 2E (component of the cornified envelope) |
