# Supplementary material for: Identification of differentially expressed genes in actinic keratosis samples treated with ingenol mebutate gel
Source: PLoS One. 2020 May 15;15(5):e0232146. doi: 10.1371/journal.pone.0232146 (PMC7228095; doi:10.1371/journal.pone.0232146)
Supplement: S3 Table — (DOCX) [file pone.0232146.s003.docx]

**S3 Table.** Gene Ontology and Reactome pathway enrichment analyses for 62 upregulated genes in post-treatment versus pre-treatment actinic keratoses that responded to ingenol mebutate gel.

|  | Biological Process (GO) | |  |
| --- | --- | --- | --- |
| Pathway | Description | FDR | Upregulated matching proteins |
| GO:0043062 | Extracellular structure organization | 1.40E-04 | ABI3BP, AGTR1, CCDC80, CD34, COL12A1, COL14A1, COL1A2, DPT, ECM2, LOX |
| GO:0030199 | Collagen fibril organization | 1.50E-04 | COL12A1, COL14A1, COL1A2, DPT, LOX |
| GO:0030198 | Extracellular matrix organization | 1.80E-03 | ABI3BP, CCDC80, COL12A1, COL14A1, COL1A2, DPT, ECM2, LOX |
| GO:0042340 | Keratan sulfate catabolic process | 3.90E-03 | OGN, OMD, PRELP |
| GO:0097435 | Supramolecular fiber organization | 6.60E-03 | COL12A1, COL14A1, COL1A2, DPT, LOX, PDE4DIP, RUFY3, TTN |
|  | **Reactome pathways** | |  |
| Pathway | Description | FDR | Upregulated matching proteins |
| HSA-2022857 | Keratan sulfate degradation | 4.20E-04 | OGN, OMD, PRELP |
| HSA-2022090 | Assembly of collagen fibrils and other multimeric structures | 1.00E-03 | COL12A1, COL14A1, COL1A2, LOX |

GO: Gene Ontology; FDR: false discovery rate.
