# Supplementary figures and images for: Identification of differentially expressed genes in actinic keratosis samples treated with ingenol mebutate gel
Source: PLoS One. 2020 May 15;15(5):e0232146. doi: 10.1371/journal.pone.0232146 (PMC7228095; doi:10.1371/journal.pone.0232146)

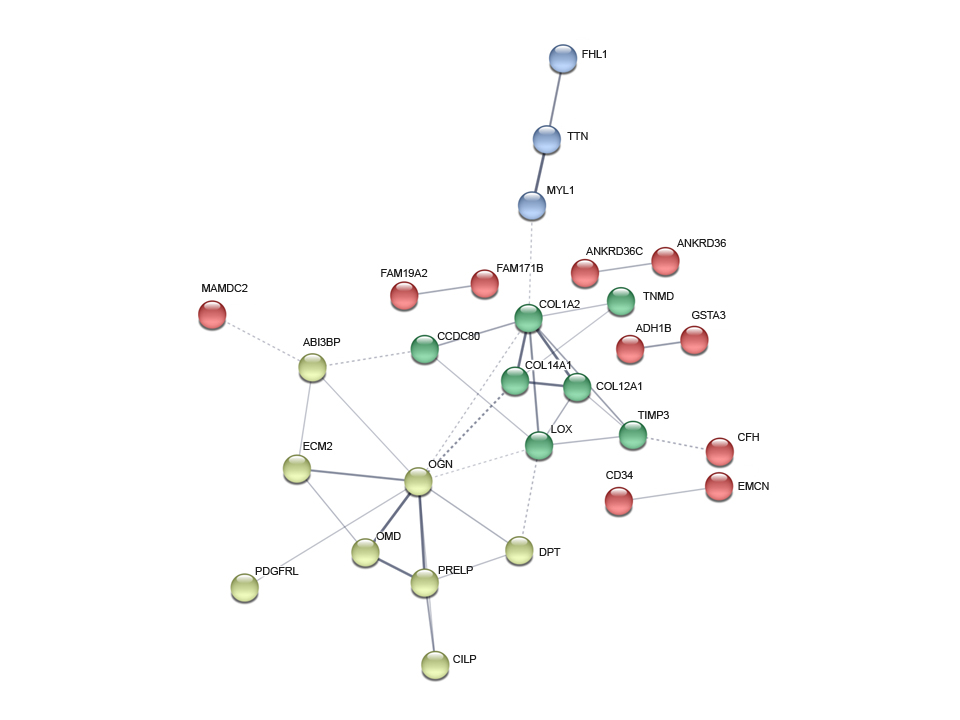

Supplement: S1 Fig — Nodes represent proteins and different line intensities denote the type of evidence for the interaction. Statistical analysis results for the network: number of nodes: 62, number of edges: 43; average node degree: 1.39; average local clustering coefficient: 0.331; expected number of edges: 7; PPI enrichment p-value < 1E-16. (TIF) [file pone.0232146.s004.tif]

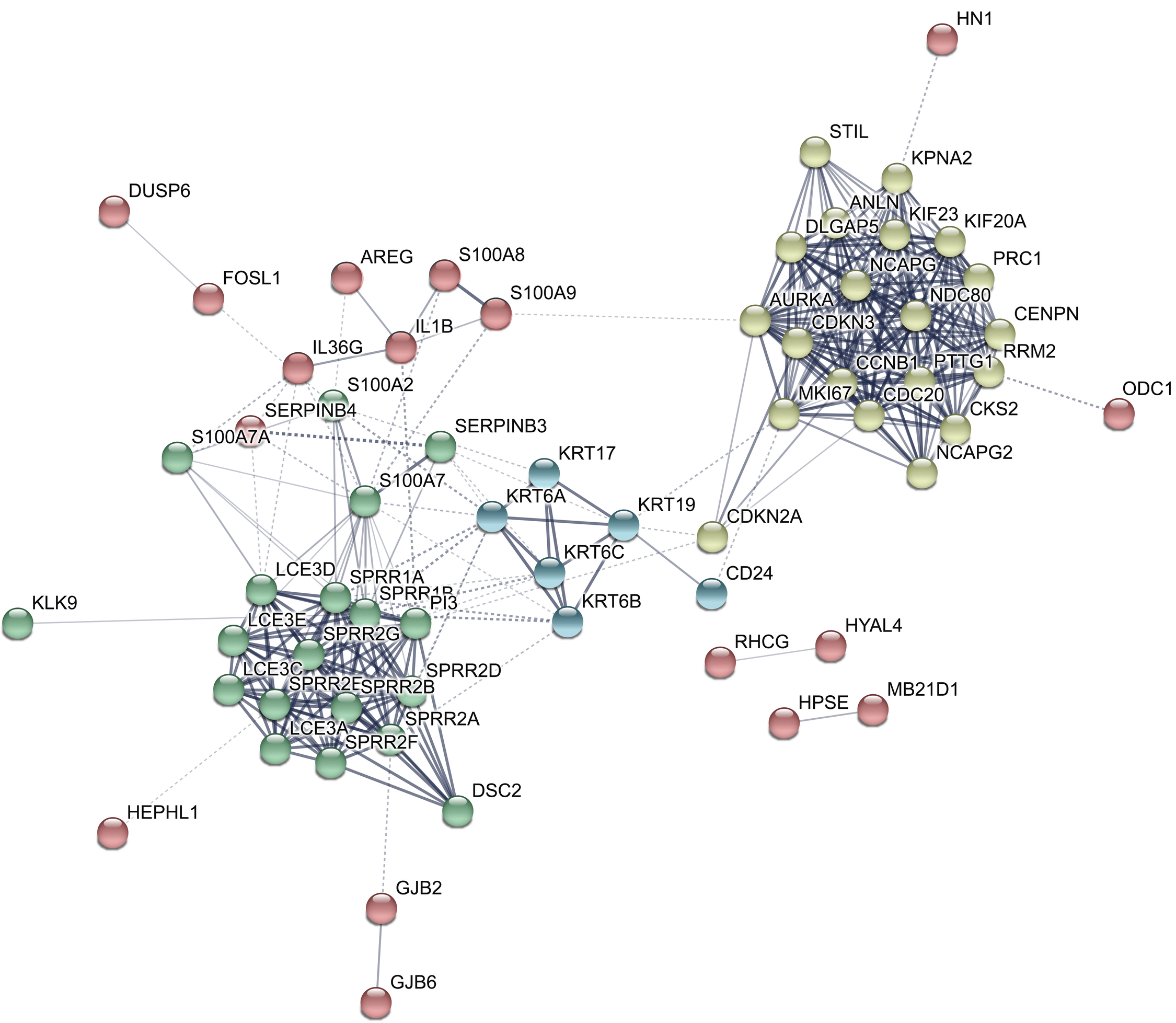

Supplement: S2 Fig — Nodes represent proteins and different line intensities denote the type of evidence for the interaction. Statistical analysis results for the network: number of nodes: 72, number of edges: 336; average node degree: 9.33; average local clustering coefficient: 0.656; expected number of edges: 39; PPI enrichment p-value < 1E-16. (TIF) [file pone.0232146.s005.tif]

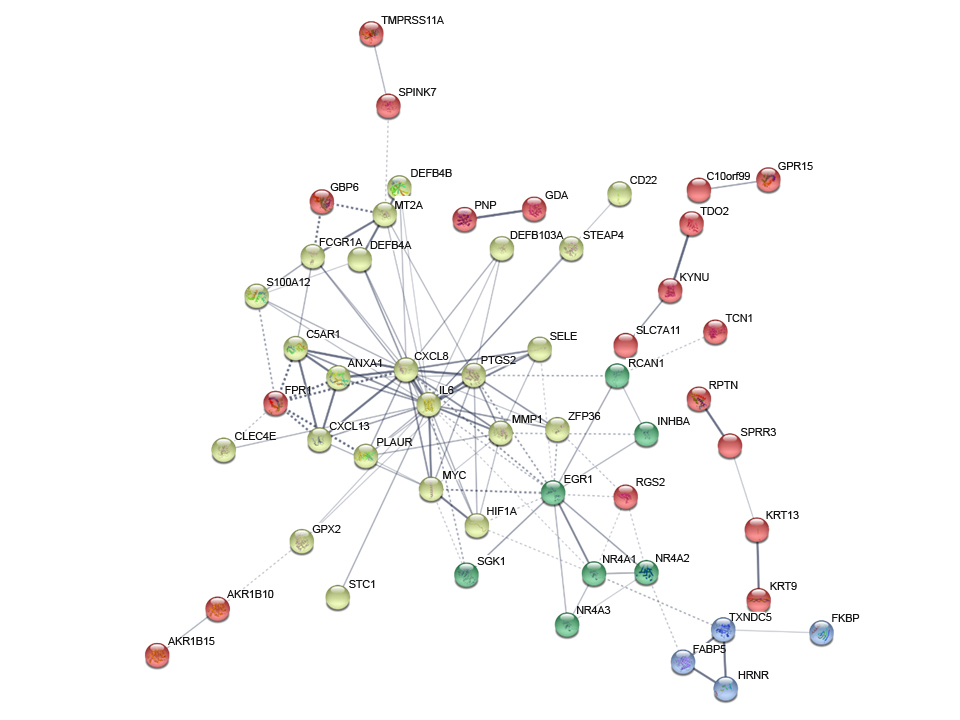

Supplement: S3 Fig — Nodes represent proteins and different line intensities denote the type of evidence for the interaction. Statistical analysis results for the network: number of nodes: 76, number of edges: 116; average node degree: 3.05; average local clustering coefficient: 0.44; expected number of edges: 37; PPI enrichment p-value < 1E-16. (TIF) [file pone.0232146.s006.tif]

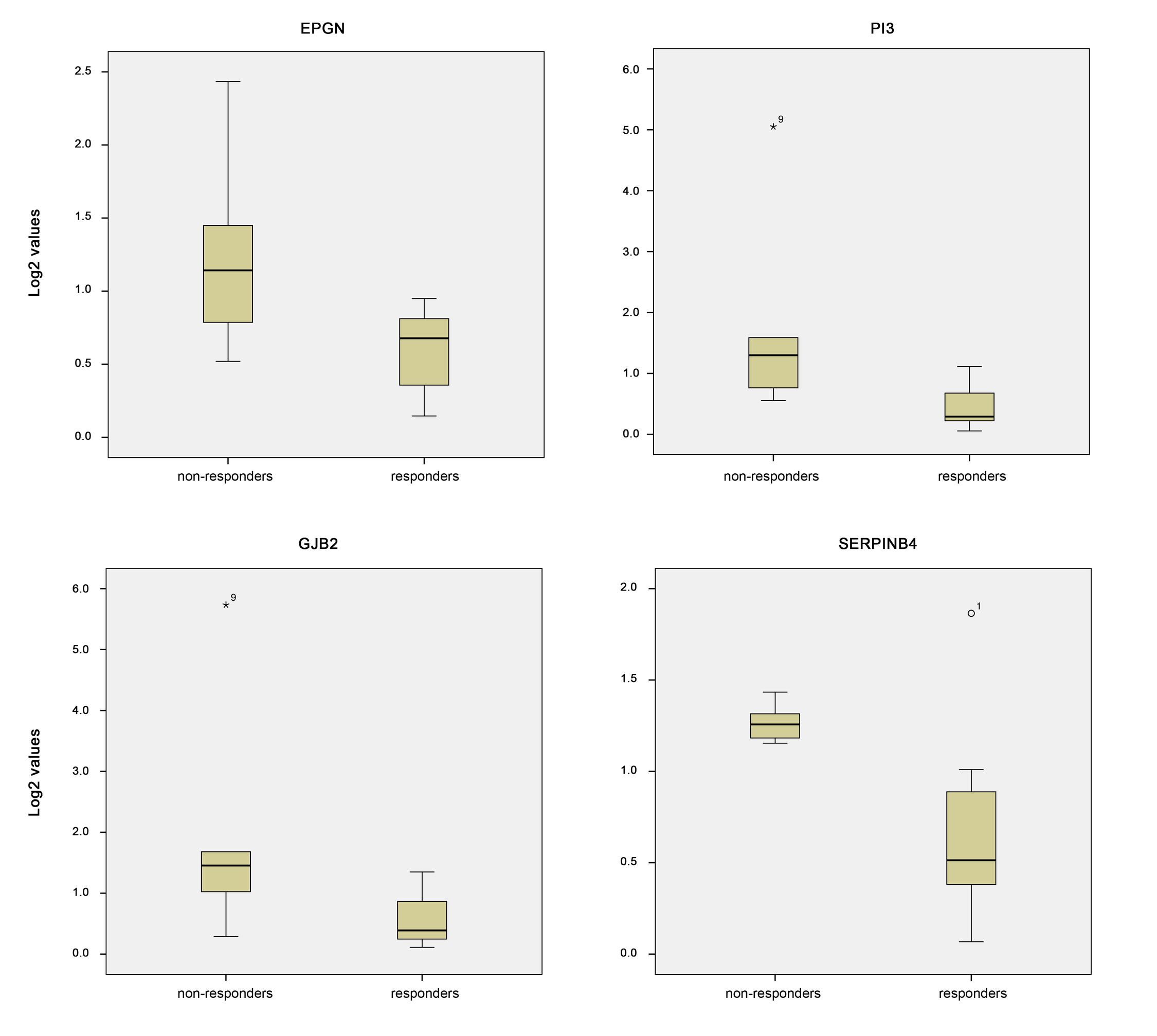

Supplement: S4 Fig — The box plots show the log2 expression levels in responder and non-responder patients (p-values <0.05). Empty circles indicate mild outliers and asterisks extreme outliers. (TIF) [file pone.0232146.s007.tif]
